# Supplementary material for: Serum Lactate Dehydrogenase Level One Week after Admission Is the Strongest Predictor of Prognosis of COVID-19: A Large Observational Study Using the COVID-19 Registry Japan
Source: Viruses. 2023 Mar 2;15(3):671. doi: 10.3390/v15030671 (PMC10058713; doi:10.3390/v15030671)
Supplement: Supplementary file 1 [file viruses-15-00671-s001.zip › viruses-2212674-supplementary.pdf]

viruses

Supplementary Table S1. Factors associated with in-hospital mortality selected for the multivariate analysis using the stepwise method (including laboratory values on day 1 *)

Variables	Estimate	Standard Error	χ2	*p*-Value
Sex	−0.4091	0.1119	13,374	<0.001
Age	0.0919	0.0053	295.152	<0.001
BMI	0.0161	0.0128	1.583	0.18
Cardiovascular diseases	0.5373	0.1409	14,547	<0.001
Respiratory diseases	0.4889	0.1385	12,454	<0.001
Liver diseases	0.6836	0.2553	7.172	0.008
Renal diseases	0.4112	0.2791	2.170	0.15
Neoplasms	0.6049	0.1531	15,610	<0.001
Diabetes mellitus	0.3371	0.1118	9.090	0.004
Cerebrovascular diseases	0.3071	0.1368	5.040	0.03
Lymphocytes_day1	−0.0409	0.0056	54,199	<0.001
Creatinine_day1	0.1471	0.0338	18,975	<0.001
Platelets_day1	−0.0020	0.0006	10,660	0.001
CK_day1	0.0001	0.0000	3.561	0.05
CRP_day1	0.0010	0.0005	3.826	0.06

* Day 1 refers to the day when the patient was admitted to the hospital. Abbreviations: BMI, body mass index; CI, confidence interval; CK, creatine kinase; CRP, C-reactive protein; LDH, lactate dehydrogenase; OR, odds ratio; WBC, white blood cell.

Supplementary Table S2 Factors associated with in-hospital mortality selected for the multivariate analysis using the stepwise method (including laboratory values on days 1 and 8)

Variables	Adjusted OR	95% CI			χ2
Sex	0.804	0.624	to	1.036	2.836
Age	1.085	1.071	to	1.100	150,602
BMI	0.972	0.944	to	1.000	3.733
Cardiovascular diseases	1.575	1.146	to	2.164	7.851
Respiratory diseases	1.562	1.144	to	2.133	7.862
Liver diseases	1.640	0.901	to	2.988	2.618
Renal diseases	1.495	0.817	to	2.734	1.700
Neoplasms	1.816	1.281	to	2.576	11,202
Diabetes mellitus	1.446	1.123	to	1.864	8.151
Cerebrovascular diseases	1.386	1.018	to	1.888	4.306
LDH_day8	1.006	1.005	to	1.007	250,747
lymphocyte_day8	0.893	0.875	to	0.911	127,419
CRP_day8	1.018	1.011	to	1.026	25,150
Platelets_day8	0.998	0.997	to	0.999	10,983
Creatinine_day1 *	1.098	1.017	to	1.186	5.679
CK_day8	1.000	0.999	to	1.000	4.739
CRP_day1	1.001	1.000	to	1.002	1.638

* Day 1 refers to the day when the patient was admitted to the hospital. Abbreviations: BMI, body mass index; CI, confidence interval; CK, creatine kinase; CRP, C-reactive protein; LDH, lactate dehydrogenase; OR, odds ratio; WBC, white blood cell.

Supplementary Table S3 Predictive characteristics of in-hospital mortality based on LDH values on admission day 8 (*n* = 8860)

LDH Threshold (U/L)	Sensitivity	Specificity	PPV	NPV	True Negative	True Positive	False Negative	False Positive
100	99.58%	0.27%	5.35%	92.00%	23	473	2	8362
150	99.37%	6.82%	5.70%	99.48%	572	472	3	7813
200	97.47%	31.10%	7.42%	99.54%	2608	463	12	5777
220	96.42%	42.52%	8.68%	99.53%	3565	458	17	4820
222	95.79%	43.32%	8.74%	99.45%	3632	455	20	4753
250	92.21%	57.89%	11.04%	99.24%	4854	438	37	3531
300	84.00%	76.31%	16.73%	98.83%	6399	399	76	1986
320	80.84%	80.94%	19.37%	98.68%	6787	384	91	1598
330	78.74%	82.85%	20.64%	98.57%	6947	374	101	1438
333	77.89%	83.59%	21.19%	98.52%	7009	370	105	1376
350	73.26%	86.44%	23.43%	98.28%	7248	348	127	1137
400	59.16%	92.52%	30.95%	97.56%	7758	281	194	627
440	50.95%	94.94%	36.34%	97.16%	7961	242	233	424
444	49.68%	95.17%	36.82%	97.09%	7980	236	239	405
660	17.68%	99.22%	56.38%	95.51%	8320	84	391	65

Abbreviations: LDH, lactate dehydrogenase; NPV, negative predictive value; PPV, positive predictive value.

Supplementary Table S4 Clinical prediction models for in-hospital mortality based on the multivariate analysis

**(A) Baseline model (excluding laboratory values from the analysis)**			
**Variables**	**Estimate**	**Standard Error**	**χ2**
Sex	−0.5693	0.1086	27,479
Age	0.0906	0.0051	310,676
BMI	0.0112	0.0126	0.781
Cardiovascular diseases	0.5097	0.1390	13,440
Respiratory diseases	0.5265	0.1354	15,117
Liver diseases	0.5982	0.2533	5579
Renal diseases	1.2967	0.2081	38,813
Neoplasms	0.6127	0.1500	16,671
Diabetes mellitus	0.4022	0.1095	13,498
Cerebrovascular diseases	0.3138	0.1344	5452
**(B) Model incorporating laboratory values on day 8 using the stepwise method**			
**Variables**	**Estimate**	**Standard Error**	**χ2**
Sex	−0.2408	0.1297	3448
Age	0.0809	0.0066	148,767
BMI	−0.0289	0.0148	3826
Cardiovascular diseases	0.4500	0.1628	7634
Respiratory diseases	0.4208	0.1596	6948
Liver diseases	0.4781	0.3058	2446
Renal diseases	0.6267	0.2778	5090
Neoplasms	0.5977	0.1782	11,249
Diabetes mellitus	0.3983	0.1290	9530
Cerebrovascular diseases	0.3262	0.1576	4285
LDH on day 8	0.0061	0.0004	249,514
Lymphocytes on day 8	−0.1145	0.0100	130,211
CRP on day 8	0.0185	0.0035	27,868
Platelet count on day 8	−0.0019	0.0005	11,916
CK on day 8	−0.0003	0.0002	4942

Creatinine day 8	0.0487	0.0309	2487
**(C) Baseline model plus LDH on day 8**			
**Variables**	**Estimate**	**Standard Error**	**χ2**
Sex	−0.4235	0.1238	11,710
Age	0.0932	0.0062	224,880
BMI	−0.0305	0.0149	4211
Cardiovascular diseases	0.5624	0.1552	13,126
Respiratory diseases	0.4887	0.1533	10,163
Liver diseases	0.3732	0.3057	1491
Renal diseases	1.2710	0.2366	28,858
Neoplasms	0.6276	0.1730	13,162
Diabetes mellitus	0.4443	0.1253	12,574
Cerebrovascular diseases	0.2911	0.1517	3679
LDH on day 8	0.0082	0.0004	501,267
**(D) Baseline model plus lymphocyte fraction on day 8**			
**Variables**	**Estimate**	**Standard Error**	**χ2**
Sex	−0.2723	0.1171	5410
Age	0.0741	0.0058	164,609
BMI	0.0042	0.0132	0.102
Cardiovascular diseases	0.3971	0.1507	6948
Respiratory diseases	0.4307	0.1456	8750
Liver diseases	0.6336	0.2713	5452
Renal diseases	0.6490	0.2224	8515
Neoplasms	0.5871	0.1614	13,242
Diabetes mellitus	0.3232	0.1168	7651
Cerebrovascular diseases	0.3431	0.1437	5698
Lymphocyte day 8	−0.1751	0.0098	322,203

The day of hospitalization is set as day 1. Abbreviations: BMI, body mass index; CK, creatine kinase; CRP, C-reactive protein; LDH, lactate dehydrogenase; WBC, white blood cell.
